# Randomized pilot trial of the “Mom Power” trauma- and attachment-informed multi-family group intervention in treating and preventing postpartum symptoms of depression among a health disparity sample

**DOI:** 10.3389/fpsyt.2023.1048511

**Published:** 2023-09-05

**Authors:** Jennifer M. Jester, Jessica L. Riggs, Rena A. Menke, Emily Alfafara, Meriam Issa, Maria Muzik, Katherine L. Rosenblum

**Affiliations:** Department of Psychiatry, School of Medicine, Michigan Medicine, University of Michigan, Ann Arbor, MI, United States

**Keywords:** intervention, prevention, group therapy, postpartum depression, parenting

## Abstract

**Introduction:**

Perinatal depression, a common complication related to childbearing, impacts mothers, children, and families. Efficacious interventions reduce perinatal depression symptoms; effort is needed to prevent the onset of perinatal depression. To determine feasibility and preliminary efficacy in reducing perinatal depression, we conducted a community-based, randomized parallel open pilot trial of Mom Power, a group-based intervention to improve mental health and parenting in mothers with young children.

**Methods:**

Mom Power consists of 10 group sessions, focused on parenting, child development and self-care and three individual sessions, to build rapport and provide personalized referrals. Control group participants received psychoeducational mailings. Computer-based urn randomization assigned mothers with experiences of interpersonal violence, depression, or other traumatic experiences to Mom Power (68) or control (54).

**Results:**

At 3-months post-treatment, the 31 retained women assigned to Mom Power were half as likely to meet criteria for probable depression (26%) as the 22 women retained in the control group (55%), with treatment predicting lower incidence of probable depression (OR = 0.13, *p* = 0.015). Moreover, among the 23 women who did not meet criteria for depression diagnosis at baseline, no women in the treatment group developed depression (*n* = 0, 0%) compared to control group women (*n* = 3, 30%). Logistic regression controlling for selective attrition confirmed the treatment effect on preventing new onset of depression (OR = 0.029, *p* = 0.012).

**Conclusion:**

These findings support the use of Mom Power for both treatment and prevention of perinatal depression.

**Clinical trial registration:**

https://classic.clinicaltrials.gov/ct2/show/NCT01554215, NCT01554215.

## Introduction

Perinatal depression is one of the most common health complications related to childbearing (1), impacting mothers, infants/toddlers, and families (2). It is with an estimated 6–15% of women experience perinatal depression (3–5), with higher rates among mothers with fewer economic resources (6), fewer social supports (7), history of depression or other psychiatric comorbidity (7, 8), childhood traumatization (9, 10) and intimate partner violence (11). Perinatal depression negatively impacts early bonding with the infant (12), parenting strategies (13), and child development (14, 15) and leads to increased parental stress (16), and for some, increased suicidality and death (17). Meta-analysis suggests that there are effective interventions for reducing postpartum (1, 4, 5). Psychotherapeutic interventions have demonstrated a medium effect in targeting depression with interpersonal psychotherapy having the largest effect sizes, followed by cognitive behavioral therapy and psychopharmacological treatments (1, 5). Depression interventions addressing trauma may be needed to offset increased risk of trauma and stress during the perinatal period. Rates of postpartum depression remain high despite availability of evidence-based treatment; therefore it may be equally important to focus on *prevention* of postpartum depression (2). The focus of this study was to provide data to estimate the parameters, including sample size, required to design a definitive future RCT of a manualized, multi-family, trauma- and attachment-informed, group-based intervention [Mom Power (18, 19)]; for both reducing rates and preventing onset of maternal postpartum depression. The primary objectives were to (1) measure differences between treatment and control groups in the percentage of participants reporting probable depression in the clinical range at the 3-month follow-up and (2) measure retention at the 3-month follow-up time point.

The Mom Power intervention aims to improve parenting competence, parenting skill, maternal self-care and maternal mental health. The group process incorporates elements of trauma-informed care and solution-focused therapy and aims to create a safe, responsive setting to engage participants. Elements of evidence-based interventions, including cognitive behavioral therapy, motivational interviewing, and dialectical behavioral therapy target depression and other mental health symptoms. The parenting intervention is driven by attachment theory with a primary goal of strengthening responsive parenting and the parent–child relationship (18–20). Mom Power has been evaluated across a number of settings, including outpatient clinics (19), and with military families (21).

Phase I pilot trial of the Mom Power intervention demonstrated reductions in depression symptoms and probable depression diagnosis (18), with mothers who completed the intervention (i.e., attended >70% of group sessions) reporting a reduction in depression symptoms, while non-completers did not. In a subsequent randomized controlled trial evaluating Mom Power, there were significant reductions in depressive symptoms immediately following treatment in the treated group, only for those women who entered treatment with histories of interpersonal trauma (19, 20).

Participation in Mom Power has resulted in improvements in other areas related to perinatal depression, such as comorbid psychiatric illness or parenting challenges. For example, Mom Power completers were less likely to meet criteria for PTSD immediately post-treatment (18, 19). Mothers also reported lower levels of parenting helplessness, whereas non-completers did not (18), suggesting improvements in parenting abilities. In addition, role-reversal, meaning children had an increased tendency to act as caregivers to their parents, increased for those in the control condition, but not those assigned to treatment in the pilot RCT (19). Mom Power participation was also associated with improvements in positive parenting behaviors (21), parenting reflectivity, and increases in balanced parenting representations (20).

Taken together, there is evidence that the Mom Power intervention can reduce maternal depression symptoms, particularly for those at highest risk (18, 20), and can improve parenting outcomes that are routinely linked with perinatal depression in the extant literature. The present study aims to further this line of research by examining the 3-month outcomes of the previously published RCT to determine the preliminary efficacy of participation in the Mom Power intervention on both *prevention* and *reduction* of maternal probable depression diagnosis. While prior studies have reported immediate post-treatment effects (19), there is uncertainty regarding longer effects of treatment and the feasibility of retaining sufficient numbers of participants for evaluation of longer lasting effects. This pilot trial examines intervention effects on probable depression 3 months after treatment ended to understand longer impacts of treatment. The ability to recruit and retain enough women to provide preliminary positive results of efficacy in this pilot trial will provide the rationale for performing a sufficiently powered future RCT.

## Method

This study was approved by the University of Michigan Institutional Review Board (ID no. HUM00018944).

### Participants

Women were recruited from September 2011 to May 2012, using self-referral through flyers posted in community sites, such as WIC offices and women’s shelters, or by active recruitment by primary care or mental health providers. Referring clinicians assessed mothers endorsement of at least one of the following: (1) mother’s history of childhood maltreatment or adult interpersonal violence, (2) past or current depression or anxiety diagnosis, (3) involvement with child protective services, (4) self-perceived or provider-rated social isolation, or (5) limited resources including low income, housing issues or food insecurity. In addition, women needed to be at least 15 years of age, English-speaking and have a child age 0 to 5 years old or be pregnant with a first child. A total of 122 women completed the baseline assessment during a home visit. Research staff enrolled participants, collected informed consent, and called women to inform them of their group assignment. Random assignment was made using urn randomization stratified by baseline trauma exposure and depression symptoms, with block sizes of 10–15. This ensured equal groups at baseline on the key measures of trauma exposure and depression symptoms. Control condition participants were mailed parenting information weekly [for more details, see (19)]. A sample size of 50 per arm for a pilot trial would be optimal to detect an extra small effect size [Cohen’s d = 0.1 (22)]. A sample size of 122 for the pilot trial allowed for 22% attrition. This was a parallel arm trial: 68 women were randomly assigned to the Mom Power treatment condition 45 women (66%) attended at least 7 of the 10 Mom Power group intervention sessions. In the control condition, participants were mailed parenting information weekly; 54 women were assigned to the control condition and 42 (78%) indicated that they had read at least 7 of the 10 weeks of material. At the post-treatment follow-up, 77 women completed the in-home assessments.

**Figure 1:**
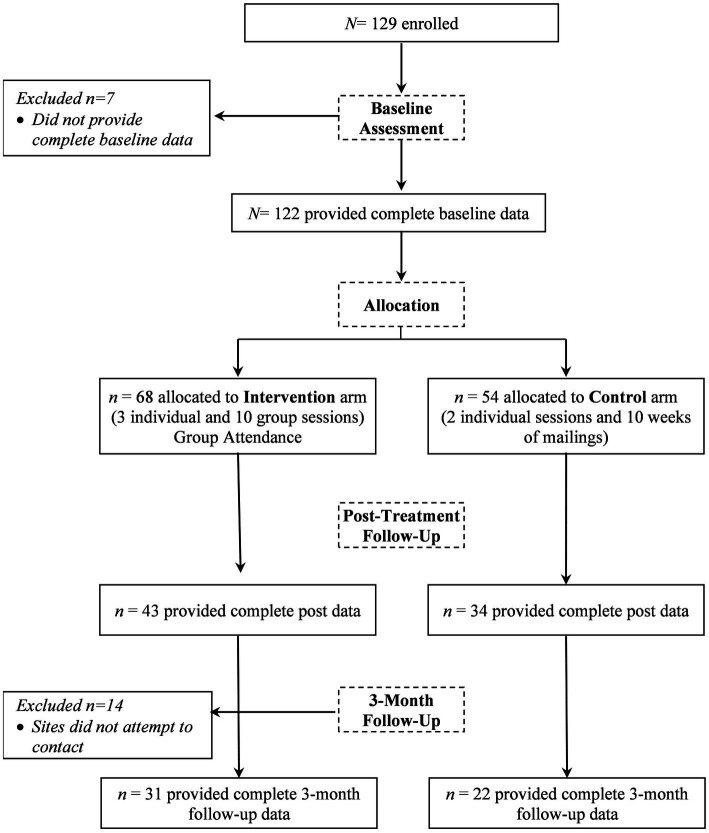
Flow diagram for the study.

The current study utilized data collected at baseline and at the 3-month post-treatment visit (see Figure 1 for study flow). At this visit, study personnel attempted to assess 108 of the original 122 women and 53 (49%) of those women completed assessments. Because of the high level of attrition, we tested and controlled for selective attrition; details are given below.

### Mom power: intervention structure

Mom Power is a 13-session (10 group session, 3 individual session) manualized intervention (19), that supports maternal mental health and the parent–child relationship using trauma-informed, infant mental health, and evidence-based methods (e.g., cognitive behavioral therapy, dialectical behavior therapy, mindfulness, and motivational interviewing). The 10 group sessions are led by master’s level clinicians that have completed a multi-day training and receive ongoing reflective consultation during the group. The target population for the group includes women with a history of maltreatment, interpersonal violence, and mental health challenges that may impact parenting. Group sessions begin with a shared meal between group facilitators, children, and mothers, during which participants and facilitators discuss a “high” and “low” from the previous week, and they may ask questions related to parenting support needs. Following the meal, there is a separation when the mothers go to another room to engage in parent-focused content. Meanwhile, the children remain with Child Team members who support the child in child-centered play, guided activities, and exploration, as well as reuniting with their mother if that is needed at any time during the group. Following separation, mothers in the group engage in a check-in, which is tailored each week to relate to topics from the previous group session. Next, Mom Power curriculum is introduced for that week. The structured curriculum includes concepts that build upon one another over time and are grounded in attachment theory. Each Mom Power session also introduces a self-care mindfulness activity for mothers in the group, including practice in group space (19). Group discussion and activities promote the parent–child relationship via strengthened knowledge of child development and responsive parenting, maternal self-care, enhancing social support, and meeting family’s material needs. Following the separate groups, mothers reunite with their children and dyads are supported in reconnecting with one another with child-friendly games, songs, and activities to promote joy in the relationship.

Individual 1-h check in sessions occur prior to, mid-way, and following the group series of sessions. The purpose of these connection points is to assess safety and tangible needs, increase rapport and build trust, identify and anticipate challenges, personalize feedback and group content, and engage in connection to other community resources that may be of benefit to the family.

### Measures

#### Maternal depression

Depression symptoms were assessed with the Postpartum Depression Screening Scale [PDSS; (23)], a self-report questionnaire comprised of 35 items that assess seven dimensions related to depression: sleeping/eating disturbances, anxiety/insecurity, emotional lability, cognitive impairment, loss of self, guilt/shame and contemplating harming oneself. Participants reported their agreement (from strongly agree to strongly disagree) with symptoms. Cronbach’s alpha for this scale was very high in this sample: 0.97 at both baseline and the 3-month follow-up. A cut-off score of 80 has been found to have a sensitivity of 94% and specificity of 98% when compared to a DSM-IV diagnostic interview (24). In a sample of women with low income in an urban setting, the sensitivity ranged from 71.2–79.5 and specificity ranged from 77.6–78.0 when compared to the Structured Clinical Interview for DSM-IV (25). Due to the high sensitivity and specificity of the cut-off scores on the PDSS, we considered scores above 80 to indicate “probable depression diagnosis.”

#### Demographic data

Demographic data, including maternal and child age, marital status, income, maternal education, and connection to public services (e.g., public insurance) was assessed at baseline using a demographic form created by the study team.

### Data analysis methods

All analysis was carried out using SAS v. 9.4.

#### Effect of treatment

Effect of treatment was estimated using logistic regression with Firth’s Penalized Likelihood, which is recommended for small samples, rare events, and situations of quasi-complete separation (26, 27). All analyses controlled for baseline level of depression symptoms.

#### Inverse probability weighting for selective attrition

For studies where missing data might be related to the outcomes of interest, inverse probability weighting can be used to control for the effects of selective attrition (28). In this study, we hypothesized that there could be selective attrition related to women’s baseline demographic characteristics and depression symptoms. Using logistic regression, we tested baseline predictors of having 3-month follow-up data, including demographic characteristics [i.e., mother’s education, age at birth of first child, race, marital status, receipt of Aid to Families with Dependent Children (AFDC)] as well as psychological measures (i.e., baseline probable depression diagnosis, worry symptoms, and history of interpersonal violence). In addition, we tested the interaction of each of these measures with the treatment group indicator. Those predictors related to having a 3-month follow-up assessment with *p* < 0.1 were retained in the final model. The final logistic regression model for attrition included baseline values of mother’s age at first birth, baseline probable depression diagnosis, and receipt of AFDC. We then found the predicted probabilities of having follow-up data from the logistic regression model. To control for selective attrition, the inverse of the predicted probability of having follow-up data was used as a weight for each participant in the models estimating treatment effects. The inverse weighting compensates for selective attrition by weighting more heavily those participants who are most likely to drop out of the study.

## Results

Table 1 shows demographic characteristics of the entire control and intervention samples at baseline and follow-up as well as the two groups separated by probable depression at baseline. At baseline, the average age of women in the program was 23.71 (SD = 8.02); more than half of the women identified as Black/African-American and 44.12% of the sample had less than a high school education. The average age of the target child was 15 months in the treatment group (79% were under 2 years of age) and 20 months in the control group (69% were under 2 years of age). Comparisons between the treatment and control groups for the entire sample and for the subsamples with and without probable depression showed that there were no differences which attained *p* value <0.1.

**Table 1:** Demographics of sample.

	Baseline		3-month follow-up		3-month follow-up No baseline probable depression		3-month follow-up Baseline probable depression	
	Treatment	Control	Treatment	Control	Treatment	Control	Treatment	Control
	*n* = 68	*n* = 54	*n* = 31	*n* = 22	*n* = 13	*n* = 10	*n* = 18	*n* = 12
	Average (sd)	Average (sd)	Average (sd)	Average (sd)	Average (sd)	Average (sd)	Average (sd)	Average (sd)
Mother age (years)	23.71 (8.02)	23.35 (5.96)	24.84 (9.30)	24.18 (5.34)	23.92 (5.32)	23.30 (8.49)	25.50 (10.02)	24.92 (5.48)
Mother age at birth of first child (years)	20.80 (5.90)	20.53 (4.49)	21.96 (7.23)	21.34 (4.02)	23.73 (8.90)	20.50 (2.42)	20.57 (5.54)	22.20 (5.16)
Age of child at baseline (months)	14.51 (13.33)	19.82 (17.17)	12.99 (12.21)	20.76 (19.47)	12.00 (8.61)	17.63 (17.29)	13.72 (14.56)	23.90 (21.90)
	*n* (%)	*n* (%)	*n* (%)	*n* (%)	*n* (%)	*n* (%)	*n* (%)	*n* (%)
Mother’s race								
African-American	42 (61.76)	28 (51.85)	18 (58.06)	12 (54.55)	10 (23.08)	6 (60.00)	8 (44.44)	6 (50.00)
White	21 (30.88)	15 (27.78)	11 (35.48)	5 (22.73)	3 (23.08)	2 (20.00)	8 (44.44)	3 (25.00)
More than one race	3 (4.41)	6 (11.11)	2 (6.45)	2 (9.09)	0 (0)	2 (20.00)	2 (11.11)	0 (0)
Latino	1 (1.47)	3 (5.56)	0 (0)	2 (9.09)	0 (0)	0 (0)	0 (0)	2 (16.67)
Native American	0 (0)	1 (1.85)	0 (0)	0 (0)	0 (0)	0 (0)	0 (0)	0 (0)
Other	1 (1.47)	1 (1.85)	0 (0)	1 (4.55)	0 (0)	0 (0)	0 (0)	1 (8.33)
Mother’s education								
Less than high school	30 (44.12)	18 (33.33)	13 (41.94)	6 (27.27)	6 (46.15)	2 (20.00)	7 (38.89)	4 (33.33)
H.S. degree or GED	9 (13.24)	11 (20.37)	4 (12.9)	5 (22.73)	1 (7.69)	2 (20.00)	3 (16.67)	3 (25.00)
Some college/voc/tech	24 (35.29)	18 (33.33)	11 (35.49)	8 (36.37)	4 (30.77)	6 (60.00)	7 (38.89)	2 (16.67)
≥ Bachelor’s degree	5 (7.35)	7 (12.96)	3 (9.68)	3 (13.64)	2 (15.38)	0 (0)	1 (5.56)	3 (25.00)
Annual household income								
Less than $5,000	23 (34.33)	14 (26.42)	12 (38.71)	6 (27.27)	6 (46.15)	3 (30.00)	6 (33.33)	3 (25.00)
$5,000 - $19,999	27 (40.3)	23 (43.4)	12 (38.75)	9 (40.91)	3 (23.08)	5 (50.00)	9 (50.00)	4 (33.33)
$20,000–$44,999	10 (14.93)	9 (16.98)	4 (12.91)	5 (22.74)	4 (30.77)	1 (10.00)	0 (0)	4 (33.33)
More than $45,000	7 (10.45)	7 (13.21)	3 (9.68)	2 (9.1)	0 (0)	1 (10.00)	3 (16.67)	1 (8.33)
Currently married								
No	53 (77.94)	39 (72.22)	23 (74.19)	16 (72.73)	10 (76.92)	8 (80.00)	13 (72.22)	8 (66.67)
Yes	15 (22.06)	15 (27.78)	8 (25.81)	6 (27.27)	3 (23.08)	2 (20.00)	5 (27.78)	4 (33.33)
AFDC								
No	61 (89.71)	48 (88.89)	30 (96.77)	21 (95.45)	10 (90.91)	21 (95.45)	18 (100)	11 (91.67)
Yes	7 (10.29)	6 (11.11)	1 (3.23)	1 (4.55)	1 (9.09)	1 (4.55)	0 (0)	1 (8.33)

Comparisons between treatment and control group, all *p* > 0.1 for each group. GED = is a series of four subject tests demonstrating high school academic knowledge. H.S., High school diploma. AFDC, Aid to Families with Dependent Children.

**Figure 2:**
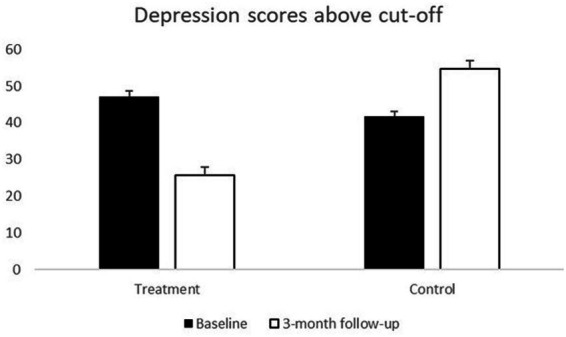
Percentage of women scoring above the cutoff for probable depression diagnosis at baseline and 3-month follow-up.

Figure 2 shows the proportion of women who scored above the risk cut-off for probable depression on the Postpartum Depression Screening Scale (PDSS). While slightly more women in the treatment sample scored above cutoff indicating probable depression at baseline compared to the control group, a higher proportion of those in the control group were above the cutoff at the 3-month follow-up compared to those who received Mom Power intervention. Logistic regression controlling for baseline depression symptoms and using inverse probability weights to account for selective attrition (Table 2) showed an effect of treatment (OR = 0.13, *p* = 0.0002), demonstrating that those who received the Mom Power intervention were less likely to meet criteria for probable depression, even after controlling for the effect of depression symptoms on attrition.

**Table 2:** Results of logistic regression predicting 3-month follow-up.

Entire sample	Treatment ratio (%)	Control ratio (%)	Odds ratio [95% Wald CI]	*p* value
Baseline	32/68 (47%)	23/54 (42%)	1.19 [0.58–2.45]	0.69
3-month follow-up	8/31 (26%)	12/22 (55%)	0.14 [0.050–0.39]	0.0002
Without probable depression at baseline				
3-month follow-up	0/13 (0%)	3/10 (30%)	0.29 [0.002–0.538]	0.018

CI, confidence interval.

We were also interested in whether there were preventive effects of the Mom Power intervention. Specifically, we were interested in examining rates of probable depression post-treatment data collection, among women who did not have probable depression at baseline. At baseline, 36/68 (53%) of women in the treatment group and 31/54 (57%) of women in the control group scored below the cutoff for probable depression [χ^2^ (1) = 0.24, *p* = 0.62]. Of these, three women in the control group met probable depression cutoff criteria at the 3-month follow-up, whereas no women in the treatment group met probable depression cutoffs. Logistic regression, weighting for selective attrition, showed that the treatment odds ratio was 0.029 [0.002–0.538] (*p* = 0.018), so that those in the treatment group without probable depression at baseline were less likely to develop probable depression by 3 months than those in the control group (see Table 2, bottom row).

## Discussion

Results of this randomized controlled trial demonstrate preliminary effectiveness of the 13-session Mom Power group intervention for both reducing rates of probable depression and preventing later onset of postpartum probable depression. Specifically, participation in Mom Power was associated with reductions in probable depression diagnosis that were sustained 3 months following treatment, as well as prevention of the onset of probable depression 3 months after treatment ended. Of note, mothers in the attentional control condition received weekly mailers containing psychoeducational and parenting material that was covered in the intervention group, indicating that participation in the group setting, guided by a trained clinician, had a meaningful impact above and beyond receipt of parenting and self-care psychoeducational content delivered via mail.

These findings are important, as they provide additional evidence to support the use of the Mom Power intervention to reduce maternal mental health symptoms, such as depression (18–20). This work also expands on previous work by examining the lasting effects of treatment by measuring probable depression diagnoses 3 months after treatment concluded. While previously published data from this randomized controlled trial examining treatment effect immediately post-treatment found that reduction in depression symptoms occurred only for those who entered treatment with a history of relational violence (19), the current study found that treatment was associated with a reduction in probable depression 3 months after treatment ended, regardless of life experiences at the onset of group. This suggests that the Mom Power intervention may result in the *quickest* reductions in depression symptoms for those with complex, interpersonal violence histories. In these cases, participation in a group may provide a supportive space that maximizes improvement for women with relational trauma histories. However, these findings also suggest that the Mom Power intervention can result in sustained, reduced depression symptoms for participants in general with effects sustained 3 months after treatment ends.

In addition to findings related to reductions in perinatal depression symptoms for those receiving the Mom Power intervention, we also found that participation in Mom Power was associated with reduced risk of developing probable perinatal depression. Because there are high rates of perinatal depression despite availability of many evidence-based interventions, it is especially important to demonstrate how treatments for perinatal depression also work to reduce the onset or recurrence of perinatal depression (2). Although the number of mothers in the study whose symptoms worsened to the point of developing new episodes of probable depression was low across the study, it is meaningful to note that no one who was randomized to the Mom Power intervention went on to develop clinical levels of depression as measured by a perinatal depression symptom measure.

Meta-analytic reviews have consistently found that the most impactful interventions to treat perinatal depression are interpersonal psychotherapy (IPT) approaches, while other interventions like cognitive behavioral therapy (CBT) and psychopharmacologic approaches are also impactful (1, 4, 5). The Mom Power intervention pulls from many evidence-based practices within a trauma-informed, relational framework. Utilizing strategies from CBT, Dialectical Behavior Therapy (DBT), mindfulness, and motivational interviewing, group facilitators focus on reducing maternal distress and mental health symptoms, while also supporting the developing relationship mothers have with their babies, and strengthening support from community, family, and/or friends. Taken together, the findings of this study support the use of the Mom Power intervention to both reduce and prevent probable perinatal depression.

This study is not without its limitations. Although accounted for statistically in the current analyses, there was substantial attrition between pre-treatment assessments and data collected 3 months post-treatment. This was unsurprising, given the nature of the sample consisting of women with young children who have experienced depression, child protective services involvement, or other traumatic circumstances. In addition, the sample size was small, and all data was collected via self-rating scales instead of diagnostic interviews. Future studies of the Mom Power intervention should aim to replicate these findings among a larger sample, and with clinician diagnosis of Major Depressive Disorder. This study utilized a self-rating scale, and although the PDSS is a highly sensitive measure of depression (24), it is not a clinical diagnostic interview. Additionally, like many studies of perinatal mood and anxiety disorders, this study focused on experiences of mothers only. Future studies should incorporate data from other parents and caregivers, including fathers and grandparents. This should be done in relation to their own mental health symptoms, as well as the impact of their presence and mental health symptoms on maternal functioning. In addition, the study included children with a wide range of ages, although 75% were under the age of 2. Future research could restrict the age range to children under 1 year of age to more narrowly focus on the postpartum period.

Finally, the study has several strengths, beginning with the use of a randomized controlled design. Additionally, the methods used to test the effect of intervention were rigorous, taking into account both baseline levels of depression symptoms, as well as accounting for the effect of depression symptoms and other participant variables that might influence attrition rates (28). The control condition used in this study was also quite robust; participants in the control group received the same group content material via mailed packets of information, as the intervention group. For an intervention effect to be found compared to this group is a strong test of the effect of the intervention. Finally, as the providers in this study were community-based clinicians serving families through their community-based social service agency, the methodology employed for this study reflected a effectiveness-implementation hybrid (29). Delivery through the community mental health system ensured that the model being tested was true to “real world” conditions, including providers focused on delivering services (versus research), study participants who were more reflective of populations served, and conditions for implementation that rendered study findings more ecologically valid and generalizable. Yet collaboration between academic researchers and community agencies, while presenting a unique opportunity, can also present a number of challenges, including the need to harmonize across research and agency policies, addressing privacy concerns, and balancing research and clinical priorities when issues emerge. Addressing these types of challenges required frequent and open communication to navigate operational procedures, training needs, and supporting fidelity in research delivery while keeping a focus on high quality service delivery for potentially vulnerable children and families (30). While this type of approach requires intensive effort, the potential benefits are substantial. Plans for larger scale implementation of an effectiveness trial will require the university-based research team to prioritize community input at all phases of study development and implementation, and the current study provides both a strong foundation as well as a template for engaging other community based agencies in this future work.

## Data availability statement

The datasets presented in this article are not readily available at the time of publication to be in accordance with the ethical consent provided by participants. Requests to access the datasets should be directed to the corresponding author.

## Ethics statement

The studies involving human participants were reviewed and approved by Michigan Medicine Institutional Review Board, Michigan Medicine. Written informed consent to participate in this study was provided by the participants’ legal guardian/next of kin.

## Author contributions

MM and KR contributed to conception and design of the study. EA assisted with data collection, data organization, and helping with group sessions. MI assisted with database and created figures for the manuscript. JJ performed the statistical analysis. JR, RM, and JJ wrote sections of the manuscript. All authors contributed to the article and approved the submitted version.

## Funding

The research presented was supported through funds from the State of Michigan, Department of Community Health (PI: Muzik, F023865-2009 and F029321-2010); Michigan Institute for Clinical & Health Research (MICHR) (PI: Rosenblum, UL1RR024986-2010), and the Robert Wood Johnson Health & Society Scholars Program (PI: Muzik, N012918-2010).

## Conflict of interest

The authors declare that the research was conducted in the absence of any commercial or financial relationships that could be construed as a potential conflict of interest.

## Publisher’s note

All claims expressed in this article are solely those of the authors and do not necessarily represent those of their affiliated organizations, or those of the publisher, the editors and the reviewers. Any product that may be evaluated in this article, or claim that may be made by its manufacturer, is not guaranteed or endorsed by the publisher.
